# Histomorphological Comparisons and Expression Patterns of *BOLL* Gene in Sheep Testes at Different Development Stages

**DOI:** 10.3390/ani9030105

**Published:** 2019-03-21

**Authors:** Taotao Li, Xia Wang, Hongyu Zhang, Zhili Chen, Xingxu Zhao, Youji Ma

**Affiliations:** 1 College of Animal Science and Technology, Gansu Agricultural University, Lanzhou 730070, China; ttli2018@163.com (T.L.); wangxiaandzisu@163.com (X.W.); 18894312250@163.com (H.Z.); ai1781417730@163.com (Z.C.); 2 College of Veterinary Medicine, Gansu Agricultural University, Lanzhou 730070, China; zhaoxx@gsau.edu.cn

**Keywords:** sheep, *BOLL* gene, testis, spermatogenesis, histomorphology

## Abstract

**Simple Summary:**

Boule homolog, RNA binding protein (*BOLL*), an ancestral member of the *DAZ* (deleted in azoospermia) gene family, is required for testicular function, maintenance, and spermatogenesis in males. However, in sheep, little is known about the expression profiles and molecular function of *BOLL*. In In this study, obvious seminiferous tubule lumens and various spermatogenic cells, including spermatogonia, primary and secondary spermatocytes, round spermatids, elongated spermatids, and spermatozoa, were observed in the testes of sheep aged 1 year and older. Our results showed that *BOLL* was expressed exclusively in sheep testes. Moreover, significant BOLL expression at the transcript and protein levels were discovered in 1- and 2-year-old sheep testes, in comparison with testes from 0-day-, 2-month-, and 5-month-old sheep. BOLL protein was located in spermatogenic cells, ranging from primary spermatocytes to round spermatids, as well as in spermatozoa with intensive immunoexpression. A preliminary study demonstrated that the sheep *BOLL* gene is critical for meiosis and sperm maturity. This study contributes to further understanding the regulatory mechanisms of the *BOLL* gene during spermatogenesis.

**Abstract:**

*BOLL* is implicated in mammalian testicular function maintenance and spermatogenesis. To understand the expression patterns and biological functions of sheep *BOLL*, we examined the expression and immunolocalization of BOLL in the developing testes of Small-Tail Han sheep aged 0 days (D0), 2 months (2M), 5 months (5M), 1 year (1Y), and 2 years (2Y), by qPCR, Western blot, and immunohistochemistry methods. Firstly, morphological studies revealed that, in addition to spermatogonia, ordered and clear spermatocytes, as well as round and elongated spermatids and sperm, were found in the 1Y and 2Y testicular seminiferous tubules of the sheep testes, compared with the D0, 2M, and 5M testes, as analyzed by hematoxylin and eosin (H&E) staining. The diameter and area of the seminiferous tubules, epithelial thickness, and the area and perimeter of the tubule lumens gradually increased with age. *BOLL* was specifically expressed in testes and upregulation of *BOLL* transcript expression was higher in the testes of the 1Y and 2Y groups than in those of the D0, 2M, and 5M groups. Similarly, BOLL protein was expressed mainly in the 1Y and 2Y testes, ranging from primary spermatocytes to round spermatids, as well as in the spermatozoa. This study is the first demonstration that sheep *BOLL* might serve as a key regulator of the spermiogenesis involved in sperm maturity, in addition to its role as a crucial meiotic regulator.

## 1. Introduction

Spermatogenesis in mammals is a complex, continuous, and tightly coordinated physiological process that is largely regulated by many genes whose expression varies at the levels of transcription and translation during different stages [1,2,3]. The *DAZ* family genes are germ cell-specific RNA-binding proteins (RBPs) which are implicated in male spermatogenesis [4,5]. During mammalian spermatogenesis, there are many RBPs in mammalian testis that play important roles in regulating gene expression at the post-transcriptional level, in order to control differentiation of the spermatogenic cells, especially of the round spermatids [6,7]. The *DAZ* family consists of three known members, including two autosomal genes, *BOLL* and *DAZL*(*DAZ*-like), and one Y chromosome gene, *DAZ* [8,9,10,11].

*BOLL* (*Boule*), an ancestral gene of the *DAZ* family, is evolutionarily conserved in all metazoans [5,12]. Numerous studies have shown that the *BOLL* gene plays an important role in testicular function, maintenance, and spermatogenesis and that loss of the gene may cause male dyszoospermia and infertility [9,13,14,15,16]. In females, *BOLL* may regulate human embryonic stem cells to enter into meiotic phase and produce meiotic germ cells which are further induced to form ovarian follicle-like cells by expressing *DAZL* and *BOLL* with recombinant *GDF9* and *BMP15* [17]. Expression of *BOLL* mRNA is significantly decreased and BOLL protein is completely lacking in the testes of infertile men, when compared to healthy men [14,15,18]. Of note, *BOLL* expression is progressively reduced with the increasing severity of testicular failure [18]. The downregulation of *BOLL* expression in male gonads is related to its methylation degree and, consequently, with meiotic arrest and infertility. A long CpG island in the *BOLL* promoter was hypermethylated in the testes of cattle–yak hybrids (male sterility) with extremely low *BOLL* expression, compared with cattle testes [19]. For males with complete spermatogenesis, the methylation degree of the *BOLL* promoter is also inversely associated with its expression level. For example, the methylation level of the *BOLL* promoter in normal human testes, with high *BOLL* expression, is significantly lower than that in somatic tissues with lacking or extremely low *BOLL* expression [9]. The same results are reported for other mammals, such as goats [20], pigs [9], and mice [9].

The transcription of the *BOLL* gene during adult mice spermatogenesis is regulated by insulin-like growth factor 1 (IGF1), through extracellular signal-regulated kinases 1 and 2 (ERK1/2) signaling and T-independent pathways [10]. In males, deletion or mutation of *BOLL* may block production of functional sperm, thereby leading to infertility [5,14,21]. However, blocked stages of spermatogenesis are different among various species. In *Drosophila melanogaster*, for example, the mutation of *Boule* results in meiotic arrest during spermatogenesis [22]. Similar findings have been published in studies of infertility in mammalian testes from human [14,15,18] and cattle–yak [23]. However, in *Boule* null mice testes, meiotic divisions proceed normally, but spermatogenic cells undergo arrest at the subsequent stage of round spermatid, prior to deformation, and do not further differentiate into mature spermatozoa [24]. By contrast, the overexpression of *BOLL* is able to promote the development and differentiation of male germ cells. Li et al. [25] reported that the overexpression of *BOLL* in male dairy goat germline stem cells can elevate the expression of meiosis-associated genes, such as *STRA8*, *SCP3*, *CDC25A*, *CDC2,* and *VASA,* promoting meiosis and spermatogenesis. In vitro, overexpression of *BOLL* may effectively promote the transdifferentiation of goat bone marrow mesenchymal stem cells into early germ cells through spermatogenesis [26]. Additionally, experiments in male mice have proven that *Boll* is involved in regulating the formation of stress granules (SGs) in germ cells under heat stress, to protect those cells from heat-induced apoptosis [27]. SGs are an assembly of untranslated messenger ribonucleoproteins that form from cytoplasmic mRNAs stalled in translation initiation under stress conditions, such as heat, hypoxia, and oxidative stress, etc. (reviewed in [28,29]), which can inhibit pro-apoptotic factors and thereby prevent cell damage [29].

Until now, most reports on the *BOLL* gene in male mammals have focused on human, cattle and mouse, but the regulatory mechanisms are not exactly the same in different species, considering the entire range and wide variety. Sheep are an economically important livestock animal with considerable agricultural significance, however, little is known about the expression profiles and biological functions of *BOLL* in sheep testes. Accordingly, it is important to elucidate the mechanisms of sheep spermatogenesis by investigating the expression patterns and regulatory roles of *BOLL* in developing sheep testes.

## 2. Materials and Methods

### 2.1. Experimental Animals and Design

All of the animals in this investigation were managed according to the animal care and experimental procedure guidelines established by the Ministry of Science and Technology of the People’s Republic of China (Approval No. 2006-398) and the study was approved by the Animal Care Committee of Gansu Agricultural University. A total of fifteen purebred Small-Tail Han sheep, from five developmental stages—0 days old (D0; *n* = 3), 2 months old (2M; *n* = 3), 5 months old (5M; *n* = 3), 1 year old (1Y; *n* = 3), and 2 years old (2Y; *n* = 3)—were provided by Sanyang Sheep Breeding Farm (Jingtai, Gansu, China). Samples from the same section of the right testis, heart, liver, spleen, lung, kidney, and longissimus dorsi muscle were obtained from each sheep. Duplicated samples were collected for all tissues: one sample was immediately placed in liquid nitrogen and stored at −80 °C for the preparation of total RNA and protein, and the other was fixed with 4% paraformaldehyde for up to 48 h, then embedded in paraffin for hematoxylin and eosin (H&E) staining and immunohistochemistry.

### 2.2. H&E Staining

Sections from testis tissues at different developmental stages were initially stained using the H&E method, then dehydrated and dewaxed using conventional histological methods, as described by Hara et al. [30] with some modifications. Sections were observed under a microscope (Sunny Optical Technology Co. Ltd., Ningbo, China).

### 2.3. Total RNA Extraction and cDNA Synthesis

Total RNA from the selected tissue samples was extracted using TRIzol Reagent (TransGen Biotech, Beijing, China), as previously described [31]. The purity, concentration, and integrity of RNA samples were determined using a spectrophotometer (Nanodrop ND-2000, Thermo Scientific, Niederelbert, Germany) and 1% agarose gel electrophoresis, respectively. Using random and anchored oligo(dT)_18_ primers (1:5 ratio), first-strand cDNA was synthesized for each sample using 500 ng RNA, according to the manufacturer′s instructions (TransGen Biotech, Beijing, China).

### 2.4. qPCR

The *BOLL* gene was amplified (LightCycler 96 Real-Time System, Roche, Switzerland) using optimized qPCR assay conditions, specifically, 1 cycle of 94 °C for 30 s; 40 cycles of 94 °C for 5 s, 40 cycles of 60 °C for 30 s. *β-actin* was used as an internal control for the normalization of *BOLL* mRNA expression. cDNA (1 μL) was added to 20 μL of the amplification reaction system used for qPCR, comprised of 0.4 μL of forward primer, 0.4 μL of reverse primer, 10 μL of 2 × TransStart^®^ Tip Green qPCR SuperMix (TransGen Biotech, Beijing, China), and 8.2 μL of ddH_2_O. The relative expression level of *BOLL* mRNA was calculated according to the 2^−ΔΔCt^ method [32]. Each experiment was biologically replicated three times, with four technical replicates each. The *BOLL* mRNA expression results, taken from an average across tissue samples, were presented in the form of bar charts. The qPCR primers, used in the present study, are shown in Table 1.

### 2.5. Western Blot

Testicular tissues at different development stages were homogenized and lysed using a radio immunoprecipitation assay (RIPA) protein extraction kit (Solarbio, Beijing, China), according to the operating instructions. Protein concentrations within the testis samples were determined using a commercial bicinchoninic acid (BCA) Protein Assay kit (Beyotime, Shanghai, China). Twenty micrograms of the denatured protein samples were separated by 12% sodium dodecyl sulfate polyacrylamide gel electrophoresis (SDS-PAGE) and then transferred onto polyvinylidene difluoride (PVDF) blotting membranes (Beyotime, Shanghai, China). After blocking in phosphate buffered saline tween-20 (PBST) containing 5% non-fat milk, the membranes were incubated overnight at 4 °C with either rabbit anti-BOLL polyclonal antibody (1:500, Bioss, Beijing, China) or anti-beta-actin polyclonal antibody (1:1500, Bioss, Beijing, China). After washing, the membranes were incubated with goat anti-rabbit IgG/HRP antibody (1:5000, Bioss, Beijing, China). Enhanced chemiluminescence signals were visualized in an X-ray room. This experiment was biologically repeated three times. Band intensities were quantified using AlphaEaseFC software (Protein Simple, Santa Clara, CA, USA). The BOLL protein expression results, from an average across tissues, were presented as bar charts.

### 2.6. Immunohistochemistry

Immunoreactivity for the BOLL protein was visualized in 5 μm sections from testicular tissues at postnatal development stages using a Histostain™-Plus kit (Bioss, Beijing, China), as previous described [31]. In brief, the endogenous peroxidase activity from testicular sections was eliminated with 3% H_2_O_2_ and then incubated with rabbit polyclonal anti-BOLL antibody (1:120, Bioss, Beijing, China) in a wet box, overnight, at 4 °C. The negative controls were generated by replacing the primary antibody with PBS. The positive signals (brown) of the BOLL protein in sections were visualized using a DAB kit (Bioss, Beijing, China) and then observed using a Sunny EX31 biological microscope (Sunny, Ningbo, China). This experiment was biologically replicated three times.

### 2.7. Image Analysis and Data Statistics

H&E staining and immunohistochemistry images for testicular cross sections were captured using ImageView software (Sunny, Ningbo, China). The morphological parameters of 35 randomly selected seminiferous tubules from the 200× magnification of H&E sections were measured using MvImage software (Sunny, Ningbo, China). The integral optical density of immunostaining for the BOLL protein was calculated by analyzing four random 400× microscope magnification levels in independently replicated sections using Image-Pro Plus 6.0 software (Media Cybernetics, Rockville, MD, USA). The data were statistically analyzed using one-way analysis of variance, with *p* < 0.05 considered as statistically significant. All displayed values, in the form of bar charts, were the mean ± SD.

## 3. Results

### 3.1. Comparison of Morphological Differences between Sheep Testes at Different Ages

**Figure 1:**
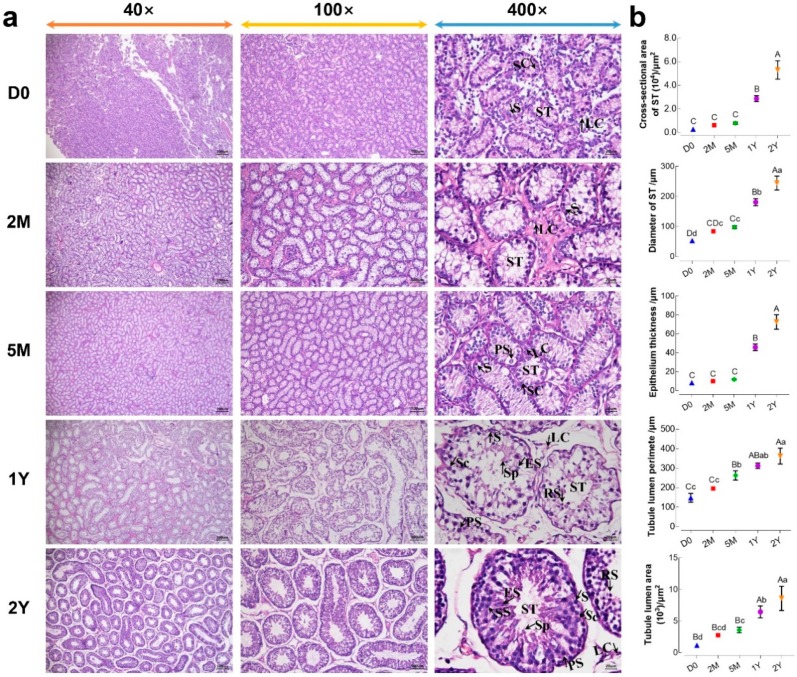
Morphological comparisons of testicular tissues at different development stages. (**a**) The representative images from D0, 2M, 5M, 1Y, and 2Y testicular cross sections at 40×, 100×, and 400× magnification, respectively; (**b**) Values of the morphological parameters obtained from the seminiferous tubules at different ages: LC, Leydig cell; SC, Sertoli cell; ST, seminiferous tubule; S, spermatogonia; PS, primary spermatocyte; SS, secondary spermatocyte; RS, round spermatid; ES, elongated spermatid; Sp, spermatozoa. Data were presented as mean ± SD in the graphs. Different capital letters denote an extremely significant difference (*p* < 0.01), while different lowercase letters denote a significant difference between groups (*p* < 0.05). D0: 0 days old; 2M: 2 months old; 5M: 5 months old; 1Y: 1 year old; 2Y: 2 years old.

The morphological observation of sheep testes at different ages, using H&E staining, were presented in Figure 1a. As the results show, testicular tissues were mainly composed of Sertoli cells and various kinds of germ cells within seminiferous tubules, as well as Leydig cells localized in the seminiferous tubule interspaces. In the D0 and 2M groups, spermatogonias were observed only in the seminiferous tubules near basement membranes. In the 5M group, a small number of primary spermatocytes were observed in the seminiferous tubules, in addition to spermatogonia. In the 1Y and 2Y groups, the spermatogenic cell layer was noticeably increased in seminiferous tubules, and various spermatogenic cells—including spermatogonia, primary spermatocytes, secondary spermatocytes, round spermatids, elongated spermatids, and sperm—were observed in an orderly and distinct arrangement. Furthermore, the morphological variables in the seminiferous tubules of the testes, at different stages of development, were summarized in Figure 1b. The cross-sectional area and diameter of the seminiferous tubules, epithelial thickness, and the area and perimeter of the tubule lumen gradually increased with age. Of note, the histomorphological parameters in the above 1Y and 2Y groups were significantly increased compared to those in the D0, 2M and 5M groups (*p* < 0.05).

### 3.2. Expression Patterns of the BOLL Gene at the Different Developmental Stages of Sheep Testes

**Figure 2:**
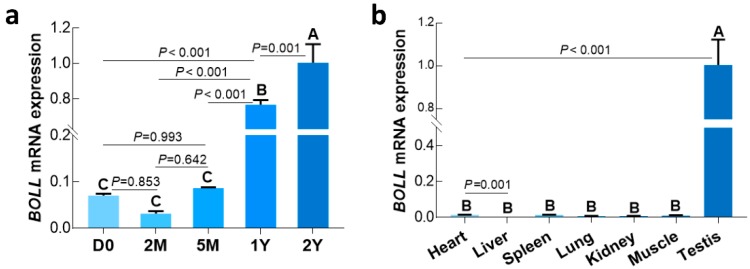
Relative expression of *BOLL* mRNA in selected sheep tissues detected by qPCR. (**a**) *BOLL* mRNA expression in sheep testis tissues at different development stages; (**b**) *BOLL* mRNA expression in testis and somatic tissues from 1Y sheep. *β*-*actin* was used as a reference gene. All experiments were biologically replicated three times, each with four technical replicates. The bars represent the mean values ± SD of 12 replicate samples obtained from three sheep per group. Different capital letters denote an extremely significant difference between groups (*p* < 0.01). D0: 0 days old; 2M: 2 months old; 5M: 5 months old; 1Y: 1 year old; 2Y: 2 years old.

**Figure 3:**
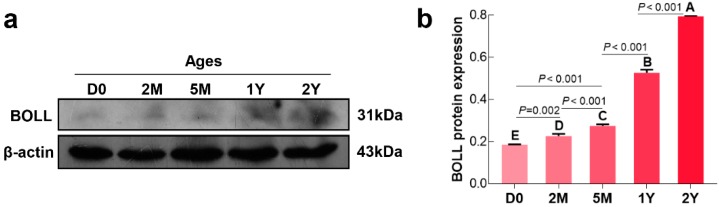
Relative BOLL protein expression in sheep testes at different ages. (**a**) A representative Western blot detection result; (**b**) The integrated density values (IDVs) of BOLL protein from β-actin was used as an internal reference protein. The experiment was biologically repeated three times. The bars represent the mean values ± SD of three replicate samples obtained from three sheep per group. Different capital letters denote an extremely significant difference between groups (*p* < 0.01): D0: 0 days old; 2M: 2 months old; 5M: 5 months old; 1Y: 1 year old; 2Y: 2 years old.

We first examined the temporal expression of *BOLL* transcript and protein in postnatal sheep testes using qPCR and Western blot, respectively. Here, we found that expression of *BOLL* was relatively low in sheep testes from the D0 to 5M groups, but was dramatically upregulated in the 1Y and 2Y groups (Figure 2a). Similarly, BOLL protein expression steadily increased in the testes from the D0 to 2Y groups, with the highest expression observed in the 2Y group (Figure 3).

### 3.3. Expression Patterns of BOLL in Various Tissues of 1-Year-Old Sheep Testes

To understand whether sheep *BOLL* was expressed in other tissues in addition to testis, we subsequently used qPCR to characterize the expression patterns of *BOLL* transcript in multiple tissues of 1-year-old sheep, including the testis, heart, liver, spleen, lung, kidney, and longissimus dorsi muscle. As expected, *BOLL* mRNA was only expressed exclusively in the testis, while no expression was detected in somatic tissues such as the heart, liver, etc. (Figure 2b).

### 3.4. Immunolocalization of BOLL Protein in Postnatal Developmental Sheep Testes

**Figure 4:**
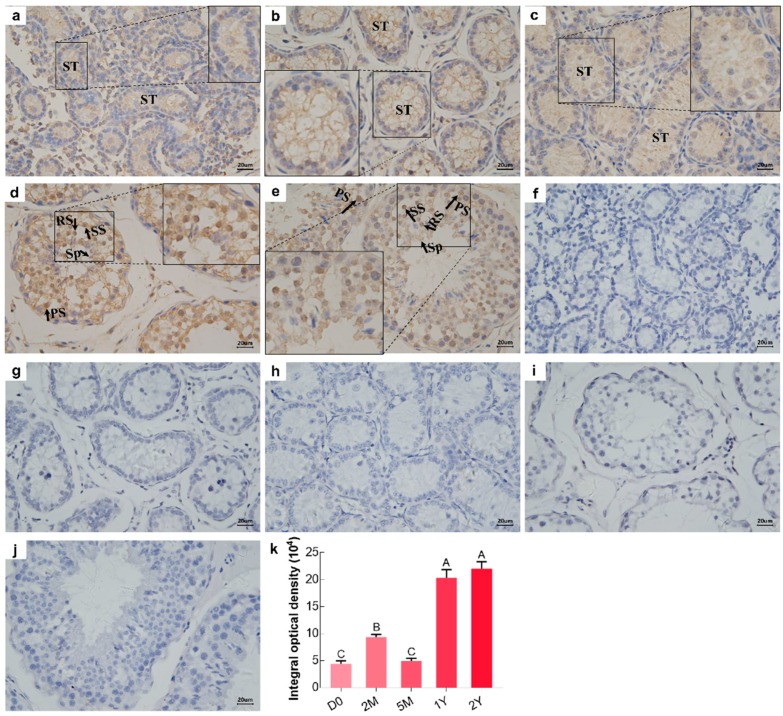
Immunohistochemical staining (*brown*) of BOLL protein in sheep testes at different developmental stages. (**a**–**e**) Immunostaining patterns of BOLL protein in the D0, 2M, 5M, 1Y, and 2Y sheep testes, respectively; (**f**–**j**) Substitution of PBS for the primary antibody served as a negative control; (**k**) Integral optical density of BOLL protein: ST, seminiferous tubule; S, spermatogonia; PS, primary spermatocyte; SS, secondary spermatocyte; RS, round spermatid; Sp, spermatozoa. The experiment was biologically replicated three times. Different capital letters denote an extremely significant difference between groups (*p* < 0.01). D0: 0 days old; 2M: 2 months old; 5M: 5 months old; 1Y: 1 year old; 2Y: 2 years old. Scale bars, 20 μm.

Immunostaining patterns for the BOLL protein in all testicular tissue sections, from sheep at different development stages, was analyzed by immunohistochemistry using a primary antibody to BOLL. The representative results are provided in Figure 4. The strength of positive reactions of the BOLL protein in testicular tissues ranged from weak to intense, increasing with age. Specifically, positive staining patterns corresponding to localization of the BOLL protein in the D0, 2M, and 5M groups were similar and showed its presence in the epithelia of seminiferous tubules, with relatively low expression (Figure 4a–c,k), whereas intense BOLL protein staining was mainly observed in the primary spermatocytes, secondary spermatocytes, round spermatids, and spermatozoa in the 1Y and 2Y groups (Figure 4d,e,k).

## 4. Discussion

Small-Tail Han sheep are an excellent genetic germplasm resource and economic breed in China. Therefore, studying sheep testicular histomorphology, as well as gene expression and regulation during testis development, has an important significance for understanding sheep fertility. In the present study, Small-Tail Han sheep testes were obtained from five postnatal developmental stages: D0 (new birth), 2M (weaning), 5M (pre-puberty), 1Y (sexual maturity), and 2Y (adult). When compared to the D0 and 2M testes, histomorphological observation showed that spermatocytes initially appeared in the seminiferous tubules beside the spermatogonia in 5M testes, indicating that Small-Tail Han sheep have an earlier age of sexual maturity. All levels of spermatogenic cells, from spermatogonia to spermatozoa, could be seen in the seminiferous tubules, most obviously in the tubules from the 1Y and 2Y testes. The measurement of the histomorphological parameters in the cross sections of testicular seminiferous tubules, at different development stages, indicated that the area and diameter of the seminiferous tubules, epithelial thickness, and the area and perimeter of the tubule lumen became gradually larger with testicular development. A significant increase was found in the 1Y and 2Y testes.

*BOLL* is a marker of germ cell development and meiosis, and previous studies in some male mammals have indicated that *BOLL* expression is restricted to the gonads and implicated in spermatogenesis through regulating spermatocyte meiosis and the male gamete formation required for fertility [5,14,33]. *BOLL* expression was examined in adult testes from cattle and yaks—low expression was detectable in testes of cattle–yaks, but there was no expression in other tissues, including the epididymis, kidney, spleen, stomach, hypothalamus, and pituitary tissues from cattle, yak, and cattle–yak [34]. In addition, two alternative splice variants of *Boule*, namely *Boule1* and *Boule2,* were also found to be exclusively expressed in yak testes, while no expression was observed in other examined tissues, including ovary, muscle, kidney, and spleen [23]. Similarly, testis-specific expression for *BOLL* is also well-documented in male humans [9], dairy goats [25], pigs [9], mice [9] and chickens [9,33]. In addition, *BOLL* expression was only present in male and female gonads for fish such as Asian seabass (*Lates calcarifer*) [35], Chinese sturgeon (*Acipenser sinensis*) [36], and medaka (*Oryzias latipes*) [37]. In this study, *BOLL* was specifically expressed in the testes of 1-year-old sheep, but was completely lacking in other male somatic tissues, such as heart, liver, spleen, lung, kidney, and muscle, as analyzed by qPCR, which is consistent with previous studies on cattle [34], goats [25], and mice [9]. The results suggest a function for *BOLL* in sheep testes which might be related to its previously reported role in testicular development or spermatogenesis.

The expression patterns and regulation mechanisms of *BOLL* are not the same in the testes of different species nor in the same species at different development stages. In mice, Zhang et al. [9] report that *Boll* expression is barely detectable from postnatal 3- to 14-day-old testes, but its expression was significantly upregulated at 12-days-old and later. In goats, the expression of *BOLL* in the testes was observed to be lacking, or low, during the embryonic and pre-pubertal stages, but gradually increased during postnatal progression with a significant expression in mature testes [25,26]. Moreover, *BOLL* expression in adult dairy goat testes with complete spermatogenesis was significantly higher than that in testes with azoospermia or male intersex [25], which demonstrates that *BOLL* is crucial for male fertility. Herein, we first examined the expression patterns of the *BOLL* gene at the mRNA and protein level during postnatal sheep testis development, as analyzed using qPCR and Western blot. Consistent with previous studies [9,26], *BOLL* mRNA and protein expression were observed to be at extremely low levels in sheep testes from the D0, 2M, and 5M groups, but dramatically increased expression was detectable in the 1Y and 2Y groups. One can speculate that the *BOLL* gene is crucial for the testicular development of postnatal sheep and, particularly, for post-pubertal sheep.

*BOLL* is a testis-specific gene that regulates spermatogenesis in males, but its distribution in the testis varies between different species and between different development stages. Positive BOLL protein is restricted to spermatocytes in the testes of normal adult men, but it is completely lacking in the testes of infertile men, which is reported by Luetjens et al. [14]. For primates, positive BOLL protein is originally observed in zygotene spermatocytes, reaching its maximal expression in pachytene spermatocytes. It is also found in the secondary spermatocytes and earlier round spermatids of pygmy chimpanzee and gray mouse lemur testes, but it is distributed only in pachytene spermatocytes in common marmoset testis [38]. In adult mice, immunostaining of the BOLL protein has been observed in spermatocytes and round spermatids, as reported by two previous papers [10,27]. Also, the same results are reported in adult goat testes [26]. Moreover, positive BOLL cells were mainly located in primary spermatocytes, with a relatively low immunoexpression in secondary spermatocytes in the adult testes of Asian seabass [35] and medaka [37]. To understand the patterns of localization of BOLL-positive cells in developing sheep testes, BOLL protein immunoreactions were further assessed using immunohistochemistry. As a result, BOLL protein was detectable in sheep testes throughout different stages of development, with weak expression in the epithelia of seminiferous tubules from new birth to the pre-pubertal stages of development, but with intense expression in primary spermatocytes, secondary spermatocytes, round spermatids, and sperm from the post-pubertal developmental stage of sheep testes. High immunoexpression patterns in testicular spermatocytes and round spermatids of post-pubertal sheep were basically in agreement with previous studies that examined fertile men [14], primates (except the common marmoset) [38], goats [26], and mice [10,27]. In contrast to previously published studies, we also observed the presence of BOLL protein in sperm from the seminiferous tubules of post-pubertal testes, which may be due to the different species as well as developmental stages investigated in this study. Taken together, these results suggest that *BOLL* might play several important roles in the meiotic phase and spermiogenesis of mature sheep.

## 5. Conclusions

In conclusion, our study investigated the expression patterns and cellular localization of *BOLL*, an ancestral gene of the *DAZ* family, in sheep testes at different ages. Our data showed that *BOLL* demonstrates an extremely low level of expression and is confined to the seminiferous epithelium in testes, from birth through to the pre-pubertal stages. However, its expression demonstrates a significant upregulation in post-pubertal testes, with strong positive signals observed in the spermatocytes, round spermatids, and spermatozoa. These results indicate that *BOLL* plays a key role in sheep spermatogenesis, especially during meiosis and spermiogenesis. Future studies will be implemented to investigate the specific molecular mechanisms of *BOLL* gene expression during sheep spermatogenesis.

## Figures and Tables

**Table 1:** List of the primers used in qPCR.

Gene	Accession No.	Primer Sequence (5′–3′)	Product Length
*BOLL*	XM_004004798.3	F: AGCAGAGAGGAAGATGGAGACC	122 bp
		R: GGGCACTCGTTGGGTTATTC	
*β*-*actin*	NM_001009784.1	F: CTTCCAGCCTTCCTTCCTGG	180 bp
		R: GCCAGGGCAGTGATCTCTTT	
